# Supplementary material for: Epidemic modelling suggests that in specific circumstances masks may become more effective when fewer contacts wear them
Source: Commun Med (Lond). 2024 Jul 7;4:134. doi: 10.1038/s43856-024-00561-4 (PMC11227579; doi:10.1038/s43856-024-00561-4)
Supplement: Supplementary file 2 — Description of Additional Supplementary Files [file 43856_2024_561_MOESM2_ESM.pdf]

Description of Additional Supplementary Files

File name- Supplementary Dataset

File description- Spreadsheet containing the data underlying figures 2,3,4
